# Culturally adaptive storytelling method to improve hypertension control in Vietnam - “We talk about our hypertension”: study protocol for a feasibility cluster-randomized controlled trial

**DOI:** 10.1186/s13063-015-1147-6

**Published:** 2016-01-14

**Authors:** Jeroan J. Allison, Hoa L. Nguyen, Duc A. Ha, Germán Chiriboga, Ha N. Ly, Hanh T. Tran, Ngoc T. Phan, Nguyen C. Vu, Minjin Kim, Robert J. Goldberg

**Affiliations:** Department of Quantitative Health Sciences, University of Massachusetts Medical School, 368 Plantation Street, Worcester, MA 01605 USA; Institute of Population, Health and Development, 18 Alley 132, Hoa Bang Street, Cau Giay District, Hanoi, Vietnam; Department of Epidemiology, Baylor Scott &White Health, 8080 N Central Expy, Dallas, TX 75206 USA; Ministry of Health, 138a Giang Vo, Ba Dinh District, Hanoi, Vietnam; Department of Pathophysiology – Immunology, Hanoi School of Public Health, 138 Giang Vo, Ba Dinh District, Hanoi, Vietnam; College of Nursing and Health Sciences, University of Massachusetts, 100 Morrissey Boulevard, Boston, MA 02125 USA

**Keywords:** Hypertension, Blood pressure, Storytelling, Trial, Vietnam

## Abstract

**Background:**

Vietnam is experiencing an epidemiologic transition with an increased prevalence of non-communicable diseases. At present, the major risk factors for cardiovascular disease (CVD) are either on the rise or at alarming levels in Vietnam; inasmuch, the burden of CVD will continue to increase in this country unless effective prevention and control measures are put in place. A national survey in 2008 found that the prevalence of hypertension (HTN) was approximately 25 % among Vietnamese adults and it increased with advancing age. Therefore, novel, large-scale, and sustainable interventions for public health education to promote engagement in the process of detecting and treating HTN in Vietnam are urgently needed.

**Methods:**

A feasibility randomized trial will be conducted in Hung Yen province, Vietnam to evaluate the feasibility and acceptability of a novel community-based intervention using the “storytelling” method to enhance the control of HTN in adults residing in four rural communities. The intervention will center on stories about living with HTN, with patients speaking in their own words. The stories will be obtained from particularly eloquent patients, or “video stars,” identified during Story Development Groups. The study will involve two phases: (i) developing a HTN intervention using the storytelling method, which is designed to empower patients to facilitate changes in their lifestyle practices, and (ii) conducting a feasibility cluster-randomized trial to investigate the feasibility, acceptability, and potential efficacy of the intervention compared with usual care in HTN control among rural residents. The trial will be conducted at four communes, and within each commune, 25 individuals 50 years or older with HTN will be enrolled in the trial resulting in a total sample size of 100 patients.

**Discussion:**

This feasibility trial will provide the necessary groundwork for a subsequent large-scale, fully powered, cluster-randomized controlled trial to test the efficacy of our novel community-based intervention. Results from the full-scale trial will provide health policy makers with practical evidence on how to combat a key risk factor for CVD using a feasible, sustainable, and cost-effective intervention that could be used as a national program for controlling HTN in Vietnam and other developing countries.

**Trial registration:**

ClinicalTrials.gov. Registration number: https://clinicaltrials.gov/ct2/show/NCT02483780(registration date June 22, 2015).

## Background

### Epidemiologic transition and cardiovascular disease (CVD)

Vietnam is in an epidemiological transition. The overall morbidity and mortality from non-communicable diseases (NCDs) has been rising rapidly over the last two decades and is a major societal problem [1]. The changing profile of chronic disease in Vietnam parallels changes in the sociodemographic characteristics of the population and increases in life expectancy [1–4]. Increased life expectancy prolongs the life-course exposure to risk factors for cardiovascular disease (CVD), rendering the population more susceptible to diseases of the heart and circulation; CVD is now the leading cause of death in Vietnam, accounting for 25 % of all deaths [5]. Concomitant with these trends, the major risk factors for CVD are either on the rise or at alarming levels in the general population. A national survey in eight Vietnamese provinces and cities found that the prevalence of hypertension (HTN) was 25 % in persons 25 years and older, and it increased with advancing age (prevalence of HTN in persons 45–54 years and 55–64 years were 42 % and 58 %, respectively) [6]. The Vietnam National Health Survey in 2002 estimated that, by 65 years of age, nearly one half of all Vietnamese men and women will have HTN [7].

### Hypertension and its impact

The World Health Organization (WHO) considers HTN to be one of the most important causes of premature mortality worldwide [8]. It is also one of the most preventable CVD risk factors; it can be easily detected and effectively treated according to evidence-based guidelines with low-cost drugs [9, 10]. In spite of the economic hardships that exist in Vietnam, inexpensive health care including generic medications to treat HTN are readily available. Our research team has, however, reported disconcerting results from a population-based survey of residents of Thai Nguyen province in 2011 in which only one third of persons diagnosed with HTN were aware of their condition. Furthermore, of those diagnosed with HTN, only 43 % were on treatment, and of those being treated for HTN, only 39 % had achieved appropriate control [11]. Furthermore, in a rural district in Vietnam, 83 % of participants who had high blood pressure (BP) were not aware of their HTN, and only 6 % of those with HTN were being treated [10]. These findings flow not only from inadequate training of community health care workers in effective health communication and lack of necessary skills to communicate the diagnosis of HTN, and the importance of control measures, to the patient, but also from poor knowledge of the importance and health impact of HTN in the general population. Indeed, a recent survey in Vietnam showed that 70 % of community health workers were unable to identify essential questions to be asked of a patient with HTN [12]. Likewise, a nationwide survey in 2007 found that only 23 % of participants understood what were the major risk factors for CVD, with men and women from rural areas having particularly poor knowledge about these risk factors [7]. These findings suggest a clear need for educating the Vietnamese population, especially middle-aged and older adults residing in rural areas, about CVD risk factors, the adverse health effects of HTN, and the development of acceptable and sustainable low-cost interventions to effectively prevent and treat elevated BP.

### Conceptual models

We urgently need novel, large-scale, and sustainable public-health interventions for detecting, treating, and controlling HTN in Vietnam. Narrative intervention, or “storytelling,” is a promising approach for engaging low-literacy populations in the treatment of their HTN. A previous randomized controlled trial of an interactive, multi-media storytelling intervention by our team documented a substantial benefit for BP control [13]. However, this work was carried out among inner-city African Americans in Birmingham, Alabama and some of the protocols used for this intervention may not directly apply to different settings. However, as storytelling is a central part of what makes us human, narrative interventions have the flexibility to undergo adaptation to achieve the best results in new cultural settings.

With this flexibility of design and approach, we are adapting our previous storytelling work for a rural Vietnam population. More specifically, we are seeking to develop a novel community-based intervention using the “storytelling” method to promote the control of HTN among middle-aged and older adults residing in rural communities in the Red River Delta region of Vietnam and to evaluate the feasibility and acceptability of the intervention with a feasibility cluster-randomized trial.

Described briefly, the intervention development process begins with Story Development Groups, consisting of small groups of patients who gather for a guided discussion. From these groups, particularly eloquent “video stars” are selected to tell their story, which may focus on the health consequences of uncontrolled HTN, overcoming barriers to HTN control, adherence to prescribed medication, and importance of dietary and lifestyle changes among other topics that connect the narrative directly with cultural practices that can have an effect in the control of HTN. The intervention team then develops customized interview guides, captures video footage, and packages the stories in an appropriate context along with supporting didactic material.

**Fig. 1:**
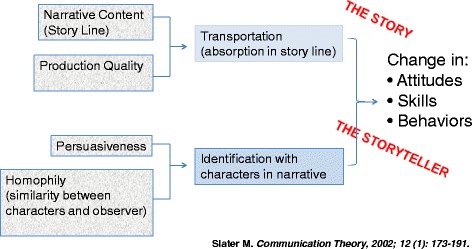
Adapted Slater Model of Narrative Communication

Storytelling is inherently culturally appropriate and well suited for populations across the literacy spectrum; it is especially well suited for populations with low health literacy [14]. Conceptually, our intervention rests on two models to empower hypertensive patients with the support of community health workers: the Health Belief Model [15] and the adapted Slater Model of Narrative Communication Theory (Fig. 1) [16].

The Health Belief Model includes four constructs representing the perceived threat and net benefits of treatment including perceived susceptibility, disease severity, benefits of treatment, and barriers. The model posits that a person is more likely to take action or change their behavior when the perceived severity and susceptibility of the disease are high and the balance between perceived barriers to changing behavior or treatment and benefits is favorable. Because HTN is primarily an asymptomatic, chronic condition that leads to serious morbidity and mortality if left untreated over several years, strategies for improving the rates of HTN control in the general population must address falsely held patient beliefs [17–19]. Our pilot data from Vietnam demonstrates a compelling lack of such awareness, and our previous work with storytelling suggests that our storytelling approach is an effective strategy for overcoming these barriers in a low-literacy population.

Narrative communication appeals to the human affinity for “storytelling.” Its effectiveness in changing attitudes and behavior follows from the ability to break down cognitive resistance through transportation (absorption in the story line) and identification with characters in the narrative. Thus, the participant is transported into the world of the characters, in this case hypertensive individuals, and becomes absorbed in the narrative content, rather than focusing on the embedded subtext of behavior change [20, 21]. The conceptual basis for the influence of stories on behavior derives from personal relevance, increased risk perception, increased self-efficacy, and transportation into the narrative (Fig. 1).

### Specific aims

The main goal of the “storytelling” intervention will be to promote engagement in preventive health care and lifestyle behaviors and improve the expected low rates of hypertension (HTN) control among adult residents of a rural province in Vietnam. The specific aims of our work are to develop and evaluate the feasibility and acceptability of a novel community-based intervention using the “storytelling” method to enhance the control of HTN in middle-aged and older adults residing in a rural province of Vietnam, which will be accomplished in several overlapping phases.

- Phase 1: developmental phase: intervention development
- Specific Aim 1: Engage community members from Hung Yen province, a rural province of Vietnam, to produce an interactive, multi-media intervention based on patients’ stories in culturally and literacy appropriate ways.
- Specific Aim 2: Produce a series of interactive digital video disc (DVDs) that encompass health message domains which are consistent with the Health Behavior Model, the Adapted Slater Model of Narrative Communication, the cultural background of participants, and the desired behavior change.
- Specific Aim 3: Use state-of-the art approaches to culturally adapt and translate intervention and assessment methods that have been successfully used in other settings and for other populations.
- Phase 2: implementation phase: feasibility trial
- Specific Aim 4: Conduct a feasibility cluster-randomized controlled trial (RCT) with four communes randomly assigned to either an intervention or comparison condition. Feasibility and acceptability outcomes will include participant engagement, recruitment, retention, intervention fidelity, feasibility of assessment procedures, and participant satisfaction.

## Methods

### Ethical statement

The Institutional Review Board at the University of Massachusetts Medical School (H00005592) and the Institute of Population, Health and Development in Hanoi, Vietnam (2014/PHAD/UMMS 01–01) approved this study.

### Study setting

“We Talk About Our Hypertension**”** will be conducted in the Red River Delta Region which is an agriculturally rich and densely populated area in northern Vietnam. In this region, communes in Hung Yen province were selected based on their general representativeness. Hung Yen province has a population of approximately 1.2 million, organized into 10 districts and 161 communes. In Vietnam, the health system is organized into four levels, namely central, provincial, district hospital, and the lowest level, which includes the community health centers (CHCs) that are responsible for providing primary health care and outpatient services. Patients with HTN are typically treated and managed at the CHCs unless they need to be referred for a higher level of care.

All communes in Hung Yen have adequate electricity. Data from a national survey in 2010 showed that 94 % of households in Hung Yen province have a television, 43 % of households have landline phones, and 37 % of the population uses mobile phones [22]. Each participant will receive a DVD player, which will be used to view DVDs, at no cost and will be given assistance in setting it up and also provided with ongoing troubleshooting.

### Phase 1. Intervention development

Intervention development will be based on successfully used protocols for story elicitation, review, editing, and packaging and tested in prior projects such as TRUST and Culturally Sensitive Intervention (CSI): Birmingham [13]. The intervention will center on stories about living with HTN, with patients speaking in their own words. The stories will be obtained from particularly eloquent patients, or “video stars,” identified during Story Development Groups. The power of this approach hinges on capturing authentic stories from real patients. In brief, the approach includes: (1) selection of “star” storytellers through Story Development Groups; (2) creation of a customized interview guide designed to elicit the most powerful stories for each star; (3) videotaping each star telling his or her story; (4) decomposition of videotaped interviews into “story units;” (5) rating of message strength for the story units by independent reviewers fluent in Vietnamese; (6) weaving the most highly rated video segments into a cohesive and authentic video production; and (7) piloting and “usability testing” [23] with multiple iterations and a “Thinking Aloud” protocol [24, 25].

The process will begin with six Story Development Groups to: (1) gather critical data to inform intervention content; (2) identify patients who will serve as our storytelling “stars”; and (3) develop customized interview guides for the subsequent videotaping of each star. Each Story Development Group will have between six and eight participants with newly diagnosed or long-standing HTN. These HTN “stars” will have had positive experiences in controlling their HTN and be particularly eloquent and persuasive advocates.

“We Talk About Our Hypertension**”** will focus on how the stars in the videos manage their HTN using positive behaviors. Digital video sequences will be rated by strength of content and emotional engagement according to our conceptual models based on adaptations of previously developed protocols from CSI: Birmingham [13]. The edited stories will be integrated into two interactive DVDs. Each DVD will have five stories on it and be about 30 minutes in length in total. The content of the DVDs will focus on a series of health message domains which are consistent with the Health Belief Model, narrative communication, the cultural background of participants, and the desired behavior change. The domains are as follows: stories about the health consequences of HTN as a silent killer, overcoming barriers to HTN control, and importance of adherence to prescribed medication, quitting smoking, dietary changes, weight loss, reducing sodium/salt intake, increasing levels of physical activity, and moderate use of alcohol, patients’ perceptions about the benefits of traditional medicine and Western medicine, and the use of traditional medicine in addition to Western medicine for managing HTN. Storytellers will discuss culturally and literacy specific aspects of patients’ perceptions of HTN and HTN treatment and deal with the daily challenges presented by achieving HTN treatment goals within the broader context of the patient’s life. In addition, the stories will be supplemented by more didactic “Learn More” content. The Learn More section will be coordinated with specific patient stories and will fill in gaps not covered by the storytellers. The Learn More section will include in lay language such topics as: What is HTN? What are the consequences of untreated HTN? How may HTN be treated without medications? What are some common medications used to treat HTN? How do these medications work and what are their side effects? Why is it important to take your medication even when you are feeling well? How should I speak to my doctor about high BP?

### Phase 2. Randomized trial evaluating the feasibility and acceptability

We will conduct a feasibility cluster-RCT with 100 adult hypertensive participants from four communes (25 participants from each commune) in Hung Yen province to assess the feasibility and acceptability of both the intervention process and the process of randomization and evaluation.

#### Study sites

In consultation with the Chief Cabinet Officer of Hung Yen province, four communes in four separate districts with a total population of approximately 12,000 men and women have been selected for this feasibility study. With a national HTN prevalence rate among adults of about 25 %, and over half of the total population being 25 years and older, the selected sites will provide enough persons with HTN for the proposed feasibility trial. Each of the selected communes have satisfied the following criteria: (1) having a CHC with a medical doctor; (2) not currently participating in other studies to improve CVD risk management; and (3) the distance between any two selected communes is more than 12 kilometers (7 miles) to minimize possible contamination.

#### Participant eligibility

To be enrolled in this feasibility study, consenting adult men and women must fulfill each of the following criteria: (1) be a resident of the selected commune; (2) be aged 50 years or older; (3) have a diagnosis of HTN according to the 7^th^ Joint National Committee of High Blood Pressure (JNC 7) [9]; (4) have BP measures consistently elevated at two time points (screening and 2 weeks after screening); (5) not be cognitively impaired (as assessed by study physicians); (6) not be a “storyteller” who was used to develop the intervention; and (7) not be a family member of another participant in the study. We will restrict our patient population to patients aged 50 years or older since their prevalence of HTN is greatest, they are interested in storytelling, and they face unique challenges to managing their HTN.

Trained study nurses at study sites will explain to eligible persons about the study’ procedures and will obtain informed consent. Patients will be excluded if they are pregnant or unable or unwilling to provide informed consent.

#### Study recruitment and randomization

Four eligible communes will be randomly assigned to either the intervention or comparison condition. Participant assignment to respective study arms will be based on the randomization status of their communes. This allocation program mirrors our plans for the future, large-scale RCT. The population at risk will be screened for HTN because available health information systems do not allow us to obtain a sampling frame at the community level. To this end, sampling frames that comprise all adult community members will be obtained under the support of community collaborators. Based on the sampling frame, commune residents 50 years and older will be randomly selected for HTN screening. To minimize selection bias, enrollment of patients will proceed in a systematic and sequential manner until the full sample size has been obtained. At the initial trial visit, researchers will explain the study protocol to possible participants and obtain informed consent. Those screening positive for HTN and not willing to participate in the study will be referred for usual care.

#### Intervention condition

After obtaining informed consent, a trained community health worker will introduce the DVD to the patient, explaining its purpose and how to use the DVD. Participants will view each intervention DVD installment at their local health center first and then engage in a post-media interview and problem-solving session with a community health worker. After the clinic viewing, the patient will take the DVD home for subsequent review and sharing with family and friends. At 3 months after randomization, the second installment of the DVD will be delivered for home viewing. After the second viewing, a study visit will be scheduled for a “post-media” interview and re-measurement of BP by a trained community health worker.

#### Comparison condition

Participants randomized to the comparison condition will receive DVDs with only didactic material about common non-communicable diseases (e.g., diabetes, heart disease, chronic lung disease – “Learn More”) but without HTN-related stories. Otherwise, the treatment and study assessments for the intervention and comparison groups will be identical. The comparison group will receive usual medical care through the CHC.

#### Translation of survey instruments

We recognize the complexity involved in cross-cultural adaptations of behavioral interventions and in translating survey instruments and data collection protocols. In adapting the study protocols and translating the data collection instruments into Vietnamese, we will follow the set of best practices developed by the United States Census Bureau [26]. According to this protocol, translation will be accomplished by a translation team, with multiple versions prepared in parallel followed by team meetings to reconcile differences.

Our translation team will consist of the two Co-Principal Investigators (PIs) from Vietnam who are fluent in the language, the PIs in the US, who bring important expertise about HTN and the intervention approach, and an expert in psychometrics. The translation process involves the following iterative steps: prepare, translate, pretest, revise, and document. In the preparation phase, the translation team will be given all relevant material to understand the purpose of the study, the intended audience, and the data collection modality and setting; translation by the Vietnam Co-PIs will proceed in parallel, with differences reconciled at team meetings. The pretesting phase will consist of cognitive interviews with five participants drawn from the local community. Cognitive testing will identify constructs specific to the Vietnamese language and culture so that appropriate adjustments may be made to ensure cross-cultural equivalence [27]. Several cycles of revision will be accomplished at full committee meetings conducted in person and by Internet video link. Each step in the process will be carefully documented. The ultimate aims of the translation process will be to produce a product that is reliable with semantic equivalence, technical accuracy and textual completeness, reads with fluency with a natural flow in Vietnamese, is appropriate to the literacy level of the intended audience, and is appropriate in style, tone, and degree of formality.

#### Data collection and management

Data will be collected at the time of baseline trial enrollment and subsequent follow-up visit at the participant’s local CHC 3 months after randomization. Each study participant’s BP will be measured at each study visit according to a standardized protocol developed by the WHO. Trained community health workers will use a calibrated Omron automated monitor to measure each participant’s BP, and the average of the last two of three readings will be entered into the database. Height and weight will be measured in the absence of shoes and heavy clothing while waist and hip size will be measured by placing the tape horizontally around the smallest part of the waist and the widest portion of the hips, respectively. Data on medication adherence will be collected using the Morisky Medication Adherence Questionnaire [28], which was specifically designed for patients with HTN. Information on patients’ use of traditional medicine for managing their HTN will also be collected.

Trained research nurses at each of the local CHCs will collect data on study participants’ sociodemographic factors and CVD risk factors including tobacco use, alcohol consumption, salt intake, and physical activity using the WHO STEPS questionnaires, which have been validated and used in previous studies examining risk factors for chronic diseases among rural Vietnamese adults [29–32]. We will ascertain self-reported engagement with the DVDs, including total viewing minutes, specific segments that were viewed, and whether the DVD was shared with family or friends. “Transportation” is a formally validated concept measuring absorption into the video narrative that has been linked to intervention effectiveness [20, 21]. Semi-structured interviews with the intervention group will solicit suggestions for refining the intervention before the larger trial. For example, participants will be asked to elaborate on what motivated or hindered their engagement with the intervention.

Data will be directly entered into a secure, password-protected, Internet-enabled, laptop computer. The computer software will have a comprehensive set of built-in quality checks, such as mandatory completion of critical fields and out-of-range flags. After each instrument has been translated, the surveys will be uploaded into REDCap, an Internet-enabled database developed by the National Institutes of Health Clinical and Translational Science Award program, which was successfully used in our previous work and which is available at no cost.

#### Study outcomes

Feasibility outcomes include recruitment, retention, engagement, and treatment fidelity. Recruitment rates will be calculated from the number of patients approached and reasons for ineligibility and non-participation. We will record the number and reasons for failure to complete the follow-up assessment. Intervention engagement, which will be mainly ascertained from the patient survey as described above, includes time spent watching the DVD, satisfaction with the viewing experience, and “transportation” into the story line. Treatment fidelity will be determined by the Vietnam Co-PIs directly monitoring 20 % of all study enrollment and follow-up visits and completing a fidelity checklist which will be carefully documented. Exploratory outcomes will include levels of systolic and diastolic BP and the proportion of participants with controlled BP defined as in JNC 8 (≤140/90 mmHg for all individuals – a different cutoff will be used for those patients ≥60 years old and do NOT have diabetes and do NOT have chronic kidney disease for whom BP should be ≤150/90 mmHg) at 3 months after randomization.

The recruitment and retention rates of study participants will be calculated for each follow-up visit in order to implement the intervention in a timely manner. Given our previous survey data about an individual’s willingness to participate in a clinical trial of HTN conducted at this study site, these rates are anticipated to be greater or equal to 80 %. Examination of our final recruitment and retention rates will allow us to focus on specific aspects of the study protocol that need to be revised. Patients’ engagement and treatment fidelity will be collected through a questionnaire survey. Based on patients’ feedback, we will revise the intervention accordingly. We anticipate that changes in BP from baseline enrollment would be 2 mmHg or greater among those in the intervention than in the control group. Based on these findings, we will carry out sample size estimates to have adequate power to detect meaningful between group differences in our prespecified major trial endpoints.

To prevent/minimize losses to follow-up and missing data, 1 week before the scheduled follow-up visit local staff will contact participants by phone as a reminder or by a home visit. Since the communes are small, it is easy to visit participants’ homes. For participants who miss the follow-up visits, study staff will come to their homes to interview and measure their BP within 2 weeks of a scheduled follow-up visit. During the course of the study, local staff will call participants every 2 weeks to find out if participants need any technical support for using DVD players, and encourage them to view the standardized DVDs more frequently to improve their adherence to the study intervention. Data collection forms are designed to be relatively short, straightforward, and culturally adapted, which will limit respondent burden and inconvenience. Finally, participants will receive a DVD player at no cost at the beginning of the study and an Omron BP monitor when they finish the study to support their initial and continued participation in the study.

#### Data analysis plan

Baseline characteristics of the intervention and usual care groups will be summarized using standard descriptive statistics. We will examine extent and mechanisms of missingness in data on each measure including participant satisfaction, recruitment, retention rates, baseline assessment, and BP. We will report numbers and reasons for recruitment and retention using a Consolidated Standards of Reporting Trials (CONSORT) diagram [33, 34] and will report results consistent with the CONSORT-EHEALTH checklist [35]. We will conduct a directed content analysis [36] of the open-ended questions to elaborate on the quantitative measures of engagement and to identify themes of satisfaction and suggestions for improvement.

Given the feasibility nature of this study, we will have limited ability to test hypotheses of intervention effectiveness. However, we will compare the distributions of systolic and diastolic BP and rates of BP control between the intervention and comparison groups. We will examine outcome variability within and between patients over time to inform the sample size calculations for the future trial. We will calculate the intraclass correlation coefficient (ICC) that accounts for the nesting of patients within commune [37–40].

#### Sample size

Leon, Davis, and Kraemer state that “power analyses should not be presented in an application for a pilot study that does not propose inferential results” [41]. Instead, we based our sample size on accepted practice for pilot studies, the considerable experience of the research team, and practical considerations [41, 42].

### Timeline

The proposed study will last 2 years. During the initial 9 months we will develop the storytelling intervention; 12 months will be subsequently devoted to conducting the feasibility trial. The final 3 months will be devoted to data analysis, report and manuscript writing, and planning the subsequent cluster-RCT.

## Discussion

We propose to develop a novel and literacy-sensitive intervention for middle-aged and older adults residing in rural communities in Vietnam. We will use a well-planned approach to culturally adapt an intervention that been effective in other settings. If our approach is ultimately proven to be successful, it will offer policy makers an innovative intervention to address a well-recognized and emerging threat to public health in Vietnam. Our approach is built on many previous “lessons learned,” and, more importantly, is low cost and of low burden to patients, clinicians, and health care systems.

However, there are some potential study caveats. The first limitation is the uptake of the intervention by patients. While the intervention has never been tested in this setting, our pilot data suggest that the intended population is interested in participating. Second are concerns with acceptance of the intervention by the CHCs. In our past experiences, we have been able to easily integrate the intervention into clinic flow. Our pilot data also suggest a willingness of clinic leaders to implement the intervention. Third, there is potential of contamination despite the distance between adjacent communes being at least 12 kilometers (7 miles). We will actively identify evidence of intervention contamination and will use this data to inform the design of our future trial. Finally, medication adherence is an important mediator of the intervention effect, but will only be measured by patient self-report.

This feasibility trial will provide the necessary groundwork for a subsequent large-scale, fully powered, cluster-RCT to test the efficacy of our novel community-based intervention. Results from the full-scale trial will provide health policy makers with practical evidence on how to combat a key risk factor for CVD using a feasible, sustainable, and cost-effective intervention that could be used as a national program for controlling HTN in Vietnam and other developing countries.

### Trial status

Participant recruitment to this trial has been completed and a total of 160 persons with HTN have been recruited to this feasibility trial.

## Abbreviations

BP: Blood pressure
CHC: Community health center
CONSORT: Consolidated Standards of Reporting Trials
CSI: Culturally Sensitive Intervention
CVD: Cardiovascular disease
DVD: Digital video disc
HTN: Hypertension
ICC: intraclass correlation coefficient
JNC: Joint National Committee
NCD: Non-communicable disease
PI: Principal Investigator
RCT: Randomized controlled trial
WHO: World Health Organization
